# Nano- and Macroscale Imaging of Cholesterol Linoleate and Human Beta Defensin 2-Induced Changes in *Pseudomonas aeruginosa* Biofilms

**DOI:** 10.3390/antibiotics10111279

**Published:** 2021-10-20

**Authors:** Brent A. Beadell, Andy Chieng, Kevin R. Parducho, Zhipeng Dai, Sam On Ho, Gary Fujii, Yixian Wang, Edith Porter

**Affiliations:** 1 Department of Biological Sciences, California State University Los Angeles, Los Angeles, CA 90032, USA; beadell@usc.edu (B.A.B.); kevin.parducho@yale.edu (K.R.P.); 2 Department of Chemistry and Biochemistry, California State University Los Angeles, Los Angeles, CA 90032, USA; andychieng98@gmail.com (A.C.); ywang184@calstatela.edu (Y.W.); 3 Molecular Express, Inc., Rancho Dominguez, CA 90220, USA; zdai@molecularexpress.com (Z.D.); sho@molecularexpress.com (S.O.H.); GFujii@molecularexpress.com (G.F.)

**Keywords:** airways, antimicrobial lipids, antimicrobial peptides, atomic force microscopy, biofilm, cholesteryl linoleate, defensins, epithelial cells, *Pseudomonas aeruginosa*

## Abstract

The biofilm production of *Pseudomonas aeruginosa* (PA) is central to establishing chronic infection in the airways in cystic fibrosis. Epithelial cells secrete an array of innate immune factors, including antimicrobial proteins and lipids, such as human beta defensin 2 (HBD2) and cholesteryl lineolate (CL), respectively, to combat colonization by pathogens. We have recently shown that HBD2 inhibits biofilm production by PA, possibly linked to interference with the transport of biofilm precursors. Considering that both HBD2 and CL are increased in airway fluids during infection, we hypothesized that CL synergizes with HBD2 in biofilm inhibition. CL was formulated in phospholipid-based liposomes (CL-PL). As measured by atomic force microscopy of single bacteria, CL-PL alone and in combination with HBD2 significantly increased bacterial surface roughness. Additionally, extracellular structures emanated from untreated bacterial cells, but not from cells treated with CL-PL and HBD2 alone and in combination. Crystal violet staining of the biofilm revealed that CL-PL combined with HBD2 effected a significant decrease of biofilm mass and increased the number of larger biofilm particles consistent with altered cohesion of formed biofilms. These data suggest that CL and HBD2 affect PA biofilm formation at the single cell and community-wide level and that the community-wide effects of CL are enhanced by HBD2. This research may inform future novel treatments for recalcitrant infections in the airways of CF patients.

## 1. Introduction

Biofilm is a mixture of microbes, extracellular polysaccharides, DNA, and proteins and promotes bacterial residence establishment and resistance to host immune factors, as well as antimicrobial drugs [1,2]. The key steps of biofilm formation are the initial attachment to epithelial cells (or indwelling devices), initially mediated by the flagella and pili, followed by growth and maturation, involving the transport of polysaccharides and proteins and the release of outer membrane vesicles into the extracellular space (and eventual dispersal) [1]. The biofilm production of *Pseudomonas aeruginosa* (PA) is central to establishing chronic infection in the airways in cystic fibrosis and contributes to treatment failure with antibiotics [3,4]. Yet, PA is not able to infect the immunocompetent host in the absence of local or systemic breaches of host defense [5]. Understanding the action of host antimicrobial factors against PA on an individual level may provide insight into the mechanisms that drive community-wide behavior during host infection, possibly leading to new forms of treatment.

On epithelial surfaces, antimicrobial peptides (AMPs) and antimicrobial lipids (AMLs)—key effector molecules of the innate immune system—play a major role in preventing colonization by microbes, such as PA [6,7,8,9]. AMPs are small amphipathic peptides with a positive net charge, due to the presence of numerous cationic amino acid residues, namely lysine and arginine [10]. AMPs can be found in vertebrates, invertebrates, plants, and fungi. Their chief antimicrobial action is pore formation on bacterial surfaces—a microbicidal mechanism, though their immunomodulatory activities are increasingly recognized [6,11]. Bacterial membranes have a more negative charge than eukaryotic membranes, which attracts the cationic AMPs to the bacterial surface, followed by membrane perturbations, mediated, in part, by the hydrophobic amino acid residues [12,13,14,15]. Furthermore, AMPs have additional intracellular effects, such as interference with DNA/RNA and protein synthesis [7]. Collectively, they exhibit potent antimicrobial activity not only against gram-positive and gram-negative bacteria, but also against fungi, protozoa, and viruses. Among the AMPs are the cysteine-rich defensins [16,17,18]. The family of defensins is subdivided based on disulfide bridge positioning and connectivity into α, β, and θ defensins. Human beta defensins are synthesized by epithelial cells and can be constitutively expressed, such as the first discovered human beta defensin HBD1, or inducible, as seen with HBD2-4 [19,20]. HBD2 has a molecular weight of ~4.3 kDa and is highly cationic, with a net charge of plus 6 and a pI of 9.2 [21]. HBD2 has previously been characterized as an AMP with chemotactic and immunomodulatory properties, in addition to its broad-spectrum bactericidal activity [22,23,24,25]. We have recently reported that at low concentrations, HBD2 inhibits biofilm formation, without reducing bacterial metabolic activity. This was accompanied by changes in the outer membrane protein profile and cell surface topology, suggesting that HBD2 induces structural changes that impair the proper function of the membrane-associated proteins involved in biofilm precursor transport into the extracellular environment [26].

While AMPs are a well-established pillar of the innate immune response, AMLs are emerging as another important host factor in combating microbes [9,27,28,29]. Cholesteryl esters, such as cholesteryl linoleate (CL), are present in respiratory epithelial cell secretions and contribute to the inherent activity of nasal fluid against PA, and liposomal formulations of CL inhibit growth of various gram-positive and gram-negative bacteria [30,31]. Furthermore, cholesteryl esters are elevated in respiratory infections and chronic inflammation, such as chronic rhinosinusitis and cystic fibrosis [8,32]. The exact route of cholesteryl ester secretion is unknown; however, one possibility is via exosomes, membrane bound vesicles that have been also implicated as transporters of other antimicrobial molecules, such as AMPs and nitric oxide [33]. The induction of both AMPs and AMLs, in response to infection in the airway mucosa, could represent a coordinated response by the epithelia, in which these immune factors augment one another’s antimicrobial activity. This is supported by the finding that the lipid fraction of nasal fluid acts synergistically with the alpha defensin human neutrophil peptide HNP-2 [30] and by the reported synergistic activity between lysozyme and docosahexaenoic acid [34] or LL-37 and vernix caseosa, the waxy coating of newborns [35].

Cholesteryl esters are highly hydrophobic and in vitro testing in aqueous solutions can be accomplished by formulating cholesteryl esters in liposomes. Liposomes are sphere-shaped vesicles that are typically composed of phospholipid (PL) bilayers [36], similar to exosomes. First described in the 1960s [37,38,39], they have been developed into a valuable drug platform for in vivo delivery of a range of compounds, with many FDA approved liposomal drugs currently on the market [40,41]. Liposomal formulations of antimicrobial drugs exhibit reduced cytotoxicity, increased activity, and improved pharmacokinetics [42,43]. We have previously demonstrated the broad-spectrum activity of liposomal formulations of various fatty acids and cholesteryl esters, including CL [31]. The ability of liposomes to deliver antibiotics inside microbial biofilms make them worth considering for treating recalcitrant biofilm-mediated infections, such as those caused by PA in cystic fibrosis [44,45,46].

In this study, we tested the hypothesis that HBD2 and CL synergistically damage the bacterial surface and disrupt the biofilm in PA, employing non-contact atomic force microscopy (AFM) and assessment of the biofilm structure after crystal violet staining, respectively. AFM has emerged as an effective in situ tool in the study of bacteria with extreme resolution [47]. Using AFM, topographical data can be obtained from individual bacteria at the nanometer scale. We found that CL containing liposomes (CL-PL) alone and in combination with HBD2 significantly increased bacterial surface roughness. Additionally, extracellular structures emanated from untreated PA, but not from PA treated with CL-PL and HBD2 alone and in combination. Crystal violet staining of biofilm revealed that CL-PL combined with HBD2 effected a significant decrease of biofilm mass and increased number of larger biofilm particles consistent with altered cohesion of formed biofilms. There is an urgent need for novel antimicrobial drugs that target biofilm formation. Dissecting the mechanism by which innate immune factors prevent biofilm formation in PA may reveal principles that could be applied to the design of novel antimicrobial agents for chronic infections involving biofilm, such as cystic fibrosis and others.

## 2. Results

### 2.1. Effects of CL-PL and CL-PL Combined with HBD2 on PA Surface at the Single Cell Level

To assess the effect of CL-PL and HBD2 on PA surface topography, we employed AFM. PACF, a well investigated strain, originally isolated from a cystic fibrosis patient [26,30,34,48], was treated for 18 h with HBD2 and CL-PL individually, combined, or incubated with the solvent control prior to processing for AFM imaging and measurement of bacterial surface roughness. To exclude curvature at the poles that would skew roughness data, we focused on the midsection of each bacterium for surface topology measurements and, in the following, we described the observed qualitative features and quantitative changes.

#### 2.1.1. AFM Demonstrates Significant Increase in Surface Roughness in PACF after Treatment with CL-PL Alone and Combined with HBD2

**Figure 1:**
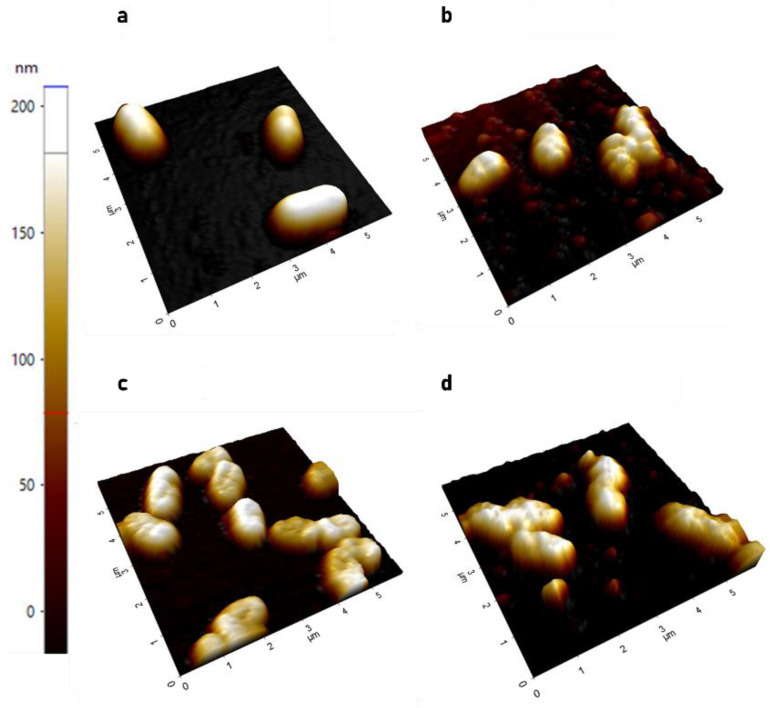
Representative three-dimensional AFM images. (**a**) Bacteria after incubation with solvent control; (**b**) CL-PL-treated bacteria; (**c**) HBD2-treated bacteria; (**d**) CL-PL + HBD2-treated bacteria. Height is depicted in a colored gradient from 0–200 nm, illustrated by the scale on the left of the figure. CL concentration was 125 µg/mL, and HBD2 concentration was 0.25 µM.

As shown in Figure 1, clear differences in the surface between the treated and non-treated bacteria are observable. CL-PL and HBD2 treatment, alone and in combination, resulted in apparently rougher bacterial surfaces than solvent control exposure. Solvent control-treated PACF (Figure 1a) appeared relatively smooth. The surface of bacteria treated with CL-PL (Figure 1b) appeared more uneven, with some round protuberances, possibly reflecting liposomal fusion with bacterial outer and/or inner membrane. HBD2-treated PACF (Figure 1c) had indentations visible on their surface that appeared to be slightly deeper in their presentation when compared to CL-PL-treated PACF. The bacteria treated with CL-PL + HBD2 (Figure 1d) appeared coarser than those treated with either CL-PL or HBD2 alone. Of note, images from CL-PL- and CL-PL + HBD2-treated bacteria also showed small, round structures, with a diameter between 50–100 nm between bacteria, most likely representing the liposomes.

**Figure 2:**
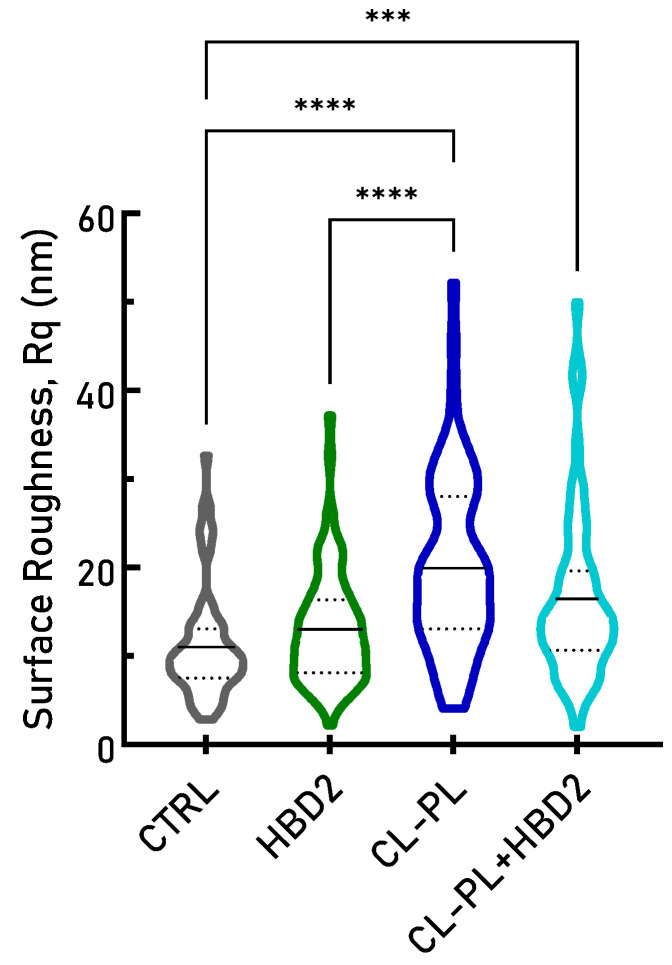
Individual bacterial surface roughness after treatment with HBD2 and CL-PL alone and in combination. PACF was incubated for 18 h in the presence of CL-PL ± HBD2 or solvent control (CTRL), followed by AFM scanning of adherent bacteria to obtain bacterial surface roughness data (derived from the central 40% of the bacterial surface, see Figure A1 for detailed calculation). CL concentration was 125 µg/mL, and HBD2 concentration was 0.25 µM. The solid line in the violin plots represents sample mean, and the dashed lines represent the first and third quartiles; *n* = 68 for CTRL, *n* = 62 for HBD2, *n* = 89 for CL-PL, and *n* = 74 for CL-PL + HBD2. *** indicates *p* < 0.001, and **** indicates *p* < 0.0001 in one-way ANOVA with Tukey’s post hoc analysis. In addition, CL-PL + HBD2 also differed significantly from CL-PL alone and HBD2 alone (*p* < 0.05).

**Figure A1:**
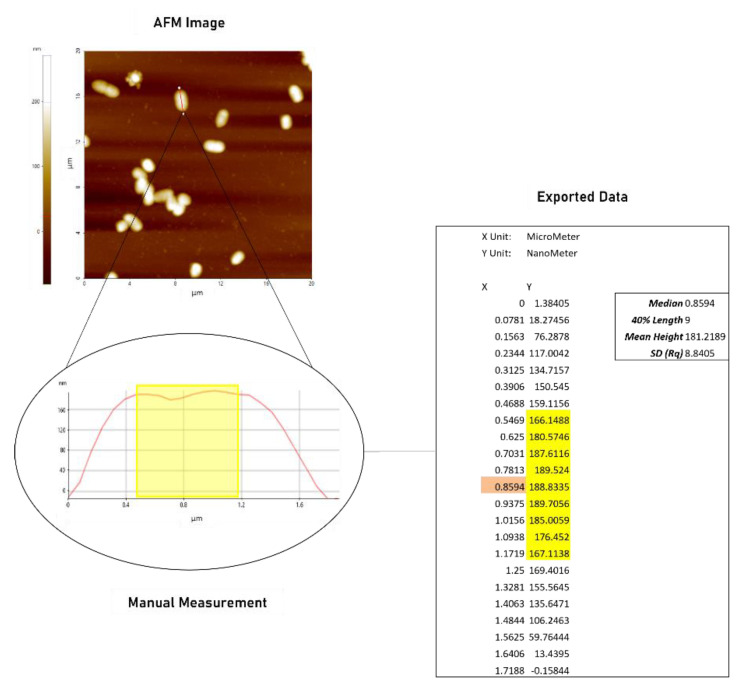
Calculation of surface roughness (Rq) for individual bacteria. After PACF was visualized with AFM, surface roughness values (Rq) were determined manually for individual bacteria. First, height data along a straight longitudinal line through the center of the bacteria were recorded and the data points falling within the central 40% of this line were further considered. The height of the data points of the 40% central line was used to calculate the average and standard deviation. The resulting standard deviation represents the Rq. Taking only the central 40% of the height data assured the Rq value are not influenced by variations of the height slopes at the poles of each bacterium.

Figure 1 represents subsections from larger 20 × 20 µm scans. To quantify the surface effects of CL-PL and HBD2 treatments, multiple 20 × 20 µm scans were taken in different areas of the adherent bacteria, followed by determination of the standard deviation of height values along the longitudinal line at the center of individual bacteria producing Rq values (see also Appendix A Figure A1). Figure 2 summarizes the Rq values from two independent experiments conducted in duplicate. Data are expressed as violin plots (see Table S1 for all data points).

Treatment with CL-PL effected a statistically significant increase in bacterial surface roughness (20.13 ± 10.01 nm, mean ± SD, *n* = 89, *p* < 0.0001), compared to the solvent control (11.09 ± 6.08 nm, mean ± SD, *n* = 68) and HBD2 (13.01 ± 6.22, mean ± SD, *n* = 62). CL-PL + HBD2 also effected a significant increase of surface roughness (16.30 ± 9.28 nm, *n* = 74), compared to the solvent control (*p* = 0.0008) and HBD2 (*p* = 0.0468), though to a lesser degree than for CL-PL alone (*p* = 0.0296). Though surface roughness appeared to be shifted to higher values for HBD2 alone, compared to the control, this trend did not reach statistical significance. These data suggest that CL-PL induces significant changes to the surface topography of PACF.

#### 2.1.2. AFM Reveals Loss of Extracellular Network Surrounding PACF after Treatment with CL-PL and HBD2 Alone and Combined

**Figure 3:**
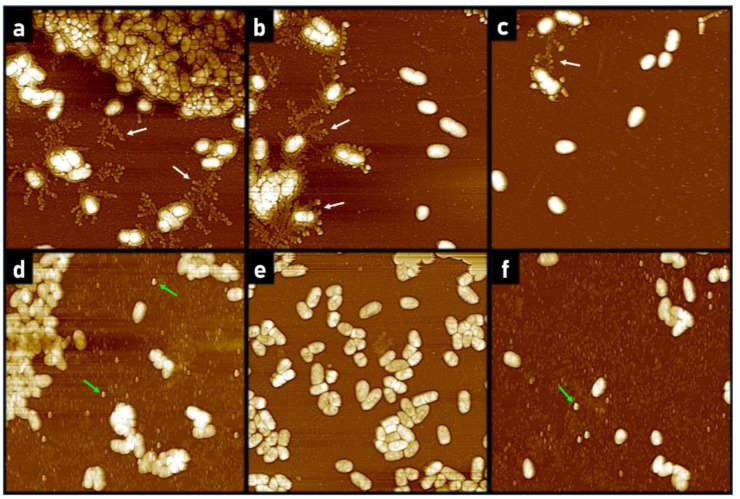
Representative 20 µm × 20 µm AFM images visualizing extracellular content surrounding individual bacteria. Bacteria were incubated for 18 h in the presence of CL-PL (125 µg/mL) ± HBD2 (0.25 µM) or solvent control. (**a**–**c**) Solvent control incubated bacteria, (**d**) CL-PL-treated, (**e**) HBD2-treated, and (**f**) CL-PL + HBD2-treated. White arrows highlight extracellular structures. The small, round structures (indicated by the green arrows) are consistent with liposomes. Images were first-order flattened. Images are representative of a total of 17 (CTRL, HBD2) or 30 (CL-PL, CL-PL + HBD2) scans per treatment, derived from two independent experiments.

While scanning the surfaces of adherent bacteria, we also noticed an alteration of the extracellular matrix surrounding the bacteria. PA and other biofilm-producing bacteria are known to produce extracellular structures early on during the biofilm formation process [2,49,50]. We observed a trend where many solvent control-treated bacteria exhibited extracellular structures that radiated outward—sometimes towards and surrounding nearby bacteria, which was absent in images from bacteria treated with CL-PL, HBD2, and CL-PL + HBD2 (Figure 3), suggesting that the organization of the biofilm matrix was interrupted by these treatments.

**Figure A2:**
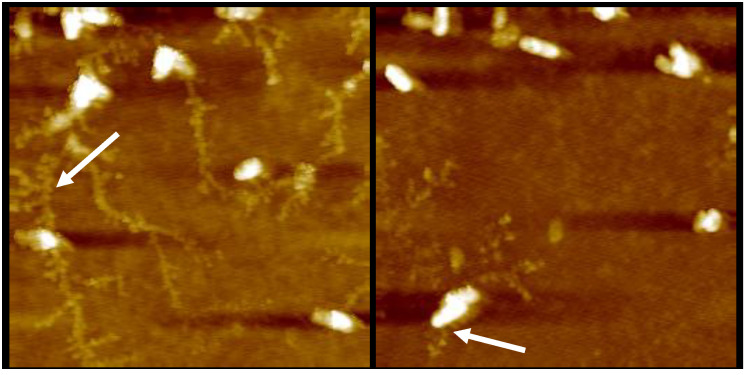
Two representative 20 µm × 20 µm AFM images visualizing extracellular content surrounding individual bacteria (PACF) after 18 h incubation in the presence of PL, liposomes without inclusion of CL. White arrows highlight extracellular structures emanating from individual bacteria. PL liposomes have a smaller diameter than CL-PL liposomes (85 nm versus 125 nm) and are not discernible in these images.

Figure 3a–c depicts control bacteria and Figure 3d–f depicts treated bacteria. In Figure 3a, a large bacterial community, with a surrounding layer of extracellular material coating, can be seen in the top of the image, with smaller clusters of bacteria nearby. The smaller clusters of PACF in Figure 3a are seen connecting to other nearby small clusters. In Figure 3b,c, PACF with and without extracellular emanating structures can be seen. Figure 3d shows PACF treated with CL-PL, and a large bacterial community can be seen without any apparent extracellular coating. Furthermore, there are no visible extracellular structures emanating from the bacteria. PACF treated with HBD2 (Figure 3e) also lacked the extracellular structures seen in Figure 3a–c, and, similarly, these structures are also missing around CL-PL + HBD2-treated bacteria (Figure 3f). The small, spherical, individual structures, of about 50–100 nm in diameter, with smooth surfaces that were only observed in samples containing CL-PL (Figure 3d,f), are most likely liposomes. Imaging from a pilot experiment indicated that the extracellular structures between individual PA were still prominent after incubation with PL liposomes only (Appendix B, Figure A2).

Thus, the AFM data supported that CL-PL alone and in combination with HBD2 induced surface damage to PA that was accompanied by a disruption of biofilm matrix production; next, we assessed biofilm production and structure at the community level after treatment with CL-PL and HBD2 alone and in combination. We employed absorbance reading and light microscopic analysis of crystal violet-stained biofilm. To further delineate CL-specific effects independent of the liposomal formulation, we also included the carrier liposomes (PL) without the addition of CL.

### 2.2. Effects of CL-PL Combined with HBD2 on PA Biofilm Production and Structure

**Figure 4:**
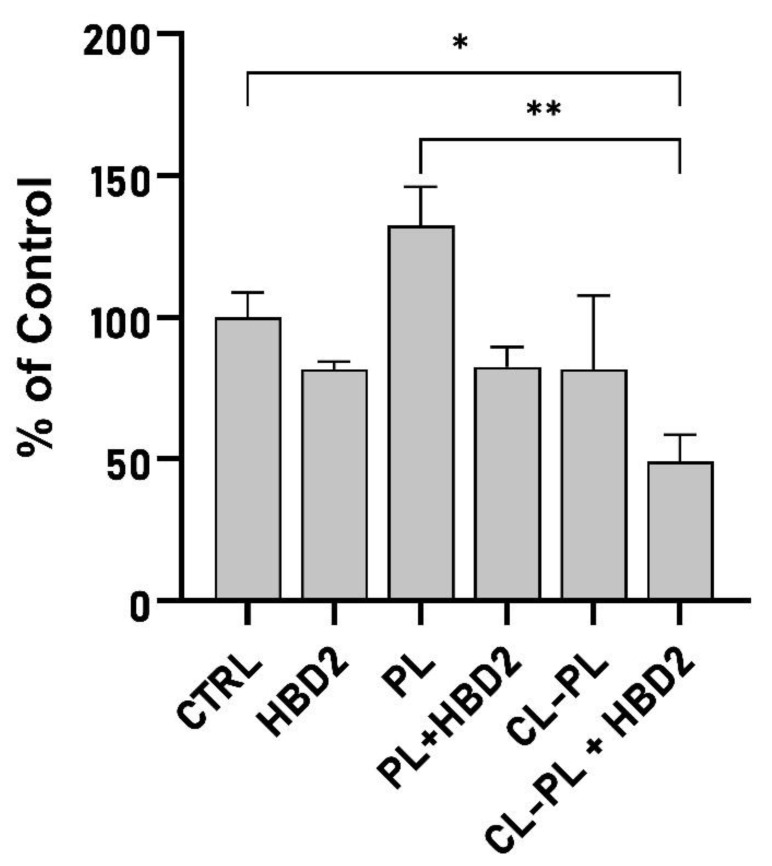
Biofilm produced by PA incubation with PL or CL-PL liposomes, in the absence and presence of HBD2. PACF was incubated in 96-well plates with the various treatments or solvent control for 18 h, after which adherent biomass was quantified with crystal violet. CTRL: solvent control; PL: liposomes composed of carrier phospholipids; CL-PL: PL with added cholesteryl linoleate. CL concentration was 125 µg/mL, and HBD2 concentration was 0.25 µM. Data are derived from two independent experiments, each conducted in replicates of 5. Data are expressed relative to the average of the solvent control. Shown are means ± SEM, *n* = 10. * indicates *p* < 0.05 and ** indicates *p* < 0.01 in Welch’s ANOVA with Dunnet’s T3 post hoc adjustment.

First, we quantified biofilm production of PACF in a microtiter format in the presence and absence of PL or CL-PL with and without HBD2. As shown in Figure 4 (means S.E.M, *n* = 10, see Table S2 for all data points), HBD2 effected a reduction of biofilm to 81.79 ± 2.64%, similar to previously reported levels [26]. PL increased biofilm formation (132.33 ± 13.74%), which could be reversed by the addition of HBD2 (82.58 ± 6.99%), leading to the levels observed for HBD2 alone. CL-PL treatment resulted in a reduction of the mean biofilm production to 81.73% of the control; however, this was accompanied by a large variation between wells (25.96%) and, thus, did not reach statistical significance. In contrast, when CL-PL was combined with HBD2, biofilm was reduced to 49.05 ± 9.52% of the control (*p* = 0.0137 in Welch’s ANOVA with Dunnett’s T3 post hoc adjustment), suggesting an, at least, additive effect between CL-PL and HBD2. Biofilm production after treatment with CL + HBD2 was also significantly reduced, compared to the biofilm production after treatment with PL (*p* = 0.0019).

**Figure 5:**
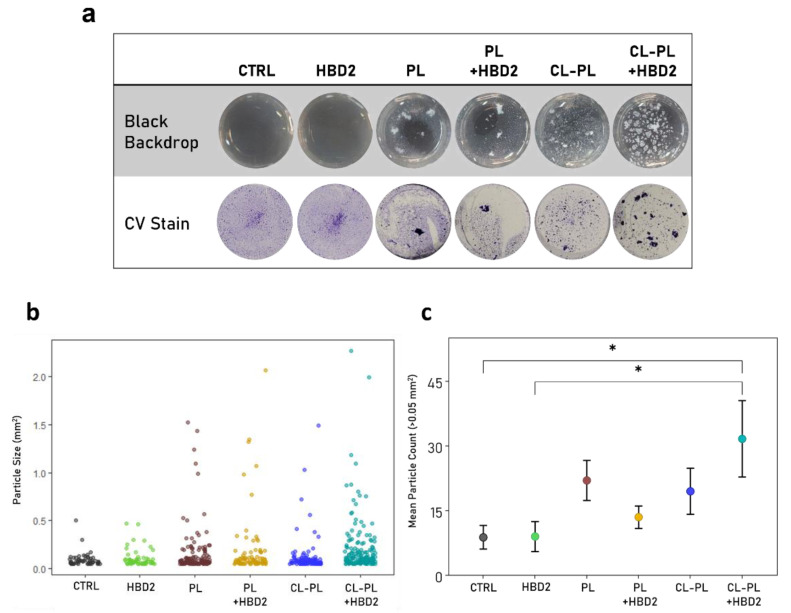
Biofilm structure of PA after treatment with HBD2 and liposomes. PA9027 was incubated for 18 h, with constant agitation, in the presence and absence of HBD2 and liposomes alone or combined in 24-well plates. CTRL: solvent control; PL: carrier liposomes; CL-PL: liposomes containing CL. HBD2 concentration was 0.25 µM; CL concentration was 125 µg/mL. (**a**) Representative images of PA9027 taken with a black backdrop before crystal violet staining (top), and after crystal violet staining, with a white backdrop. The latter images were further analyzed with ImageJ. (**b**) Distribution of stained biofilm particles over 0.05 mm^2^ in size (*n* = 101 for CTRL, *n* = 57 for HBD2, *n* = 133 for PL, *n* = 81 for PL + HBD2, *n* = 116 for CL-PL, and *n* = 182 for CL-PL + HBD2. (**c**) The number of particles above 0.05 mm^2^ per well was determined and averaged. Shown are the means ± SEM from three independent experiments performed in duplicates. * indicates *p* < 0.05 in one-way ANOVA with Tukey’s post hoc adjustment.

**Figure A3:**
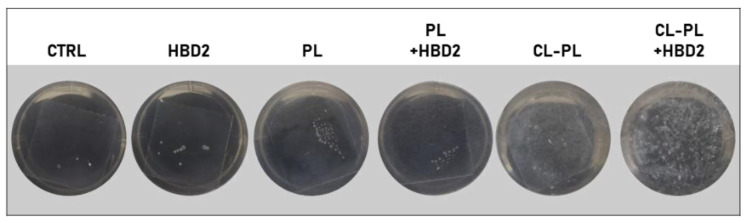
Biofilm structure of PA after treatment with HBD2 and liposomes using PACF. Bacteria were incubated for 18 h with constant agitation in the presence and absence of HBD2 and liposomes alone or combined in 24-well plates. CTRL: solvent control; PL: carrier liposomes; CL-PL: liposomes containing CL. HBD2 concentration was 0.25 µM and CL concentration was 125 µg/mL. Shown are images taken with a black backdrop.

We also noticed an uneven distribution of crystal violet staining and the appearance of numerous large crystal violet-stained aggregates in wells that had contained PACF treated with CL-PL + HBD2, which was also apparent in situ, prior to CV staining (Appendix C Figure A3). To quantify this effect (Figure 5, see Table S2 for all data points) we up-scaled the experimental setting to 24-well plates, with continuous shaking during the incubation, and took images from the biofilm in situ and after crystal violet staining (Figure 5a) and measured the size of the crystal violet-stained biofilm particles (Figure 5b,c). For these experiments, a different PA strain was used, namely PA9027, to confirm that the observed effects were not strain-specific.

Images from the in situ observed biofilm and the biofilm after crystal violet staining showed similar findings. Incubation with solvent control and HBD2 resulted in finely dispersed biofilm covering most of the well surface with central accumulation of biomass, the latter likely due to the horizontal shaking during the incubation. Incubation with PL, carrier liposomes lacking cholesteryl linoleate, with and without the addition of HBD2 resulted in formation of one larger biomass particle and areas with more finely dispersed biofilm. Incubation with CL-PL led to the formation of larger particulate biomass, consistent with a break-up of the microbial mat and increased dispersion. In contrast, incubation with CL-PL and HBD2 combined produced numerous larger biofilm masses distinct from all other treatments. The quantitative analysis substantiated this observation. Particle size distribution (Figure 5b) was similar between solvent control (CTRL) and HBD2-treated samples. Overall, larger particles were observed in the presence of liposomes, which was most pronounced after treatment with CL-PL + HBD2. The shift to biofilm with a larger particle size was also reflected in the increase of the number of particles greater than 0.5 mm^2^ per well (Figure 5c): CTRL: 8.83 ± 2.50, HBD2: 9 ± 3.18, PL: 22 ± 4.24, CL-PL: 19.5 ± 4.89, PL + HBD2: 13.5 ± 2.38, CL-PL + HBD2: 31.67 ± 8.07, means ± SEM, *n* = 6. However, compared to the solvent control (*p* < 0.05), only the treatment CL-PL + HBD2 resulted in the formation of significantly higher counts of particles larger than 0.05 mm^2^, suggesting that HBD2 and CL-PL acted together to disrupt biofilm formation in PA. 

Taken together, AFM revealed that, at the single cell level, with attention to the bacterial mid-section, the surface roughness of the bacteria was increased after treatment with CL-PL alone and in combination with HBD2, and both CL-PL and HBD2 alone and in combined inhibited the formation of extracellular matrix. At the community-wide level, biofilm staining with crystal violet demonstrated that treatment with CL-PL, in combination with HBD2, significantly reduced biofilm production and resulted in pronounced effects to biofilm organization, with a statistically significant increase in large particle counts, consistent with biofilm dispersion. These data support that HBD2 and CL-PL work cooperatively to disrupt PA biofilm formation at the single cell and community-wide level.

## 3. Discussion

The innate mucosal immune response is a crucial part of the overall immune function; yet, many aspects, especially the role of antimicrobial lipids and their interaction with antimicrobial peptides, are not well understood. We have recently demonstrated that, at low concentrations, the antimicrobial peptide HBD2 inhibits biofilm formation in *Pseudomonas aeruginosa*, without reducing its metabolic activity [26]. The purpose of this study was to examine the impact of HBD2, combined with the antimicrobial cholesteryl ester cholesteryl linoleate (CL, [30,31]), on biofilm formation in *Pseudomonas aeruginosa*. We employed atomic force microscopy and crystal violet staining to obtain insight on the individual bacterial cell and the community-wide level, respectively. Due to the hydrophobic nature of CL, it was necessary to formulate CL with a carrier [30,31]. We found that CL-PL alone and in combination with HBD2 significantly increased bacterial surface roughness. Additionally, extracellular structures emanated from untreated PA and PA treated with PL, but not from PA treated with CL-PL and HBD2 alone and in combination. Light microscopy imaging revealed that treatment with CL-PL combined with HBD2 effected a significantly increased number of biofilm particles greater than 0.05 mm^2^, consistent with reduced cohesion of formed biofilms.

AMPs, including defensins, are known to embed in bacterial membranes [17]. Thus, the export of biofilm precursors across the membrane could be impeded by embedding of HBD2 in the bacterial membrane, leading to impaired biofilm formation. AMLs are also likely to embed into bacterial membranes, due to their hydrophobic nature. This is supported by prior electron microscopic studies with the free fatty acid docosahexaenoic acid and PA [34], and the fusion of liposomes with the outer membrane of PA has been demonstrated previously with fluorescence-based assays [51,52]. Thus, we expected structural alterations in the bacterial surface topography after treatment with CL-PL alone and combined with HBD2 that could be resolved by AFM. AFM visualization has been used to illustrate bacterial surface-perturbing effects of AMPs and its relationship to their bactericidal activity [53,54,55,56], but studies on AMLs in this domain are lacking. We found significant increases in surface roughness after treatment with CL-PL with and without HBD2, though to a lesser extent when HBD2 was present. However, the size of liposomes likely led to more prominent alterations of the surface topography when interacting with the bacterial membrane, and structural changes initiated by the much smaller HBD2 are likely to be more subtle. The increased surface roughness with round appearance for CL-PL-treated bacteria could reflect liposomal fusion with the bacterial membrane. The incorporation of colloidal gold labeling into liposomes may help visualize liposome fusion onto the bacterial membrane in future studies [57].

The emanating extracellular structures, seen only in AFM images of solvent-treated PACF, appeared to extend outwards to other bacteria and interconnect small clusters of bacteria. They resembled previously described Psl-fiber scaffold networks that are important for proper biofilm formation and adherence [49]. The eDNA and type IV pili also play a role in the scaffold configuration, as defective eDNA release and type IV pili production in PA impedes the normal maturation of biofilm [50,58]. Thus, subsequent investigations could include exopolysaccharide extraction and characterization, as well as assessing the expression of type IV pili-associated genes [59] and release of eDNA. Alternatively, these structures could present outer membrane vesicles, which have been implicated in the formation of PA biofilm [60]. Of note, HBD2-treated bacteria also lacked these extracellular structures, highlighting that HBD2 independently induced structural changes in PA extracellular matrix, in agreement with our previous findings [26]. While beyond the scope of this study, future efforts could include AFM analyses to probe mechanical and chemical properties of the biofilm matrix, such as elasticity and stiffness [47,61,62], as well as the characterization and quantification of metabolites and precursors of biofilm [63,64].

Employing CV staining of biofilm, we found a significant decrease in adherent biofilm matrix with a combination of CL-PL and HBD2 treatment. Significant changes were also observed in the cohesion of PA biofilm structures, as reflected by the significant increase of large biofilm particles in the presence of CL-PL combined with HBD2. The alteration of the biofilm layer seen in CV staining might reflect, at least, in part, an inability of the bacteria in the community to interweave the biofilm film, for example via Psl fibers, as supported by our AFM data. While larger particles could represent larger aggregates of PA surrounded by a stronger biofilm matrix, less biofilm was able to adhere to the surface and adherence to epithelial cells precedes host invasion.

Only CL-PL combined with HBD2 resulted in the significant increase in large particle size, suggesting a synergistic action and highlighting the importance of antimicrobial lipids in the innate defense. Even though cholesteryl esters, as a group, have been identified in biofilm, specifically dental plaque [65], cholesteryl esters have not yet been implicated in anti-biofilm activity, to the best of our knowledge, and they could add to the arsenal of surfactants that airway epithelial cells release

There were some limitations in this study. The AFM and CV staining data cannot be directly compared, since the former was performed with bacteria exposed to glass surfaces and the latter with bacteria adherent to plastic surfaces, and it is known that surface properties influence biofilm formation [66]. Furthermore, glass and plastic are not equivalent with the mucosal lining of the airways, and future studies should investigate the effects of CL-PL and HBD2 on biofilm formation in vivo or ex vivo.

In summary, combination treatment of PA with CL-PL and HBD2 yielded significant increases in surface roughness, established by AFM studies and a loss of extracellular structures emanating from the bacterial cell. This was accompanied by significant effects on biofilm production and structure of the bacterial biofilm, consistent with a break-up of the microbial mat, as determined by CV stain. These data suggest that CL effects structural changes on the bacterial surface that interfere with biofilm formation, and these effects are further enhanced by HBD2. Novel therapies for patients suffering from chronic lung infection with biofilm producing bacteria may be informed by the action of these innate host defense molecules.

## 4. Materials and Methods

### 4.1. Antimicrobial Peptide Human Beta Defensin 2 (HBD2)

Lyophilized HBD2 was chemically synthesized and purified by Dr. Wuyuan Lu, University of Baltimore, Maryland, who generously provided the peptide for these studies [67]. The peptide was reconstituted in 0.01% glacial acetic acid to yield a 500 µM stock and stored at −20 °C. As solvent control for HBD2, 0.01% glacial acetic acid was used.

### 4.2. Liposome Preparation

Hydrogenated soy phosphatidylcholine (HSPC) and distearoyl-phosphatidylglycerol (DSPG) were bought from Avanti Polar Lipids, Inc. (Alabaster, AL, USA). Cholesteryl linoleate (CL) was purchased from Sigma-Aldrich (St. Louis, MO, USA). Liposomes were prepared via dry lipid film rehydration technique (42). Lyophilized lipids were dissolved separately in chloroform:ethanol 3:1 (*v*:*v*) solution to obtain working stocks of CL (MW 649.1, 5 mg/mL), HSPC (MW 783.77, 10 mg/mL), and DSPG (MW 801.06, 1 mg/mL), whereby 250 μL of HPLC grade dH_2_O of water was added to DSPG, after the addition of the organic solvent. Then, the lipid solutions were mixed in 16 × 100 mm borosilicate glass tubes to yield the desired lipid quantities, followed by flushing with N2 gas, which was performed to evaporate the solvent, while preventing oxidation of fatty acids, and desiccation under negative vacuum pressure (<5 microns Hg) for 4 days. Dry lipid films were rehydrated in aqueous phase with the addition of 10 mL 100 mM sodium phosphate, pH 7.0 (pre-warmed to 60 °C) for 10 min, followed by probe-sonication (Branson Sonifier 450 equipped with Stepped Tip 1/8” 630-0422) for 10 min at 35% amplitude output in a 60 °C water bath, in order to create a homogenized population of large unilamellar liposomes roughly 100 nm in size. Liposomes were sterile-filtered through a 0.22 µm pore size filter (Millex-GP filter with polyethersulfone membrane, Millipore Sigma, Burlington, MA, USA). Liposome size was ascertained using dynamic light scattering (Zetasizer Nano, Malvern Panalytical, Malvern, UK). Liposomes were formulated to contain phospholipids only (3.6 mg/mL HSPC/0.4 mg/mL DSPG; designated from now on as PL, sized around 85 nm diameter) and phospholipids and cholesteryl linoleate (1 mg/mL CL, 3.6 mg/mL HSPC/0.4 mg/mL DSPG; designated from now on as CL-PL, sized around 125 nm diameter). For experimentation, liposomes were diluted 8-fold, yielding the following final lipid concentrations: 450 µg/mL HSPC and 50 µg/mL DSPG ± 125 µg/mL CL. Solvent controls were subjected to identical liposome preparation techniques but without the addition of lipids and having been further diluted, similar to the liposome preparations.

### 4.3. Pseudomonas Aeruginosa

A *P. aeruginosa* strain, originally isolated from a cystic fibrosis patient (PACF, kindly obtained from Dr. M.J. Welsh, University of Iowa, Iowa City, IA, USA), that has been extensively researched in our lab [26,30,31,34] and others [48], was used for AFM experiments and some biofilm staining experiments. Mid-logarithmic growth phase cultures were prepared from snap-frozen overnight cultures. For each experiment of this study, an aliquot was briefly thawed and 750 µL was transferred into 50 mL pre-warmed TSB, followed by 3 h incubation at 37 °C at 200 rpm. The culture was centrifuged for 10 min at 805× *g* at 25 °C. The pelleted bacteria were resuspended and adjusted to 0.5 McFarland standard (equivalent to 1–2 × 10^8^ CFU/mL) in 140 mM NaCl (ambient temperature). *P. aeruginosa* ATCC 9027 (PA9027) was used for biofilm structural assessment only. Frozen aliquots of mid-logarithmic growth phase cultures, prepared in 25% glycerol (25 μL), were briefly thawed and then inoculated into TSB (2.5 mL), followed by 3 h incubation at 37 °C at 225 rpm. Bacteria were sedimented by brief centrifugation at 8000× *g* for 2 min, at RT the bacterial pellet was resuspended in 1 mL 140 mM NaCl, adjusted to 0.5 McFarland in 140 mm NaCl, and further diluted, as needed.

### 4.4. Atomic Force Microscopy

AFM was used to provide high-resolution images of structural changes to bacterial surfaces by CL and HBD2. Bacteria adjusted to McF 0.5 were diluted 1:20 in 140 mM NaCl (now 0.5–1 × 10^7^ CFU/mL) and immediately aliquoted into a 6-well, flat-bottom tissue culture treated wells that contained 22 × 22 mm^2^ glass microscope cover slips. Thereafter, buffered medium and treatment or solvent controls were added. The final assay conditions were ~1 × 10^6^ CFU/mL in 10% Mueller Hinton, supplemented with 140 mM NaCl and 12.5 mM sodium phosphate, pH 7.0, 0.25 µM HBD2, liposomes with and without 125 µg/mL CL, or the respective solvent controls. After 18 h incubation at 37 °C, the liquid content of the wells was removed and slides were placed in a fresh 6-well plate, then 2 mL of 2.5% glutaraldehyde (electron microscopy grade, TedPella, in 1× PBS) was added to chemically fix bacteria that were adhered to the coverslips. After incubation at 4 °C for 20 min, the fixative was removed by aspiration, cover slips were washed twice with 2 mL sterile MilliQ water, and then air dried. The surface topography of PACF was visualized using a Park Systems NX12 AFM. Non-contact cantilevers (PPP-NCHR, 42 N/m, 330 kHz), purchased from Park Systems, were used. For AFM imaging, multiple 20 × 20 µm scans, at 256 × 256 pixel resolution, were performed for each sample, with scan rates between 0.49–0.75 Hz. In addition, cropped images of representative bacterial clusters (roughly 25–36 µm^2^) from large scans were obtained for qualitative visual comparison. Bacterial surface roughness was acquired using the instrument software (Park Systems’ XEI) and differences between the various treatments were calculated, as shown in Appendix A, Figure A1.

### 4.5. Biofilm Staining and Assessment

Biofilm was quantified by crystal violet staining [68]. In round-bottom, 96-well polystyrene microtiter plates (Corning, Corning, NY, USA), 40 µL of washed bacteria, adjusted to ~2.5 × 10^6^ CFU/mL in 140 mM NaCl, was added to wells that contained either 10 µL of 10-fold concentrated HBD2 or 0.01% acetic acid and 50 µL of 2-fold concentrated buffer (20% Mueller Hinton broth, 168 mM NaCl), supplemented with either 25 mM sodium phosphate pH 7.0 or liposomes. Final lipid concentrations in liposome treatment wells were 450 µg/mL HSPC and 50 µg/mL DSPG ± 125 µg/mL CL. Plates were incubated at 37 °C for 18 h before quantification. Wells were gently rinsed 3 times with sterile dH_2_O and stained with 125 µL 0.1% crystal violet at ambient temperature, shielded from light for 10 min. Wells were then rinsed again (3 times) with sterile dH_2_O and air-dried for at least 30 min. Dried biofilms were then solubilized with 200 µL of 30% acetic acid and incubated at ambient temperature for 15 min before 125 µL from each well was transferred to an optically clear, flat-bottom 96-well polystyrene microtiter plates (Perkin Elmer, Waltham, MA, USA). Absorbance at 570 nm was determined using a Victor X3 Plate Reader (Perkin Elmer). Baseline absorbance values were determined for each treatment by wells without added bacteria. For biofilm staining for structural assessment assays, 200 µL of washed bacteria adjusted to ~2.5 × 10^6^ CFU/mL in 140 mM NaCl was added to 24-well, flat-bottom, non-treated polystyrene plate (Corning, Corning, NY, USA) wells that contained either 50 µL of 10-fold concentrated HBD2 or 0.01% acetic acid and 250 µL of 2-fold concentrated buffer (20% Mueller Hinton broth, 168 mM NaCl), supplemented with either 25 mM sodium phosphate pH 7.0 or liposomes. For all biofilm studies, outer wells were filled with sterile water to minimize edge effects during incubation. Final assay concentrations were the same as described above. Plates were incubated at 37 °C for 18 h at 420 rpm, after which images were taken using a Clarus 15× macro lens (Xenvo, China), with an illuminated diopter on a black backdrop. Then, wells were gently rinsed 3 times with sterile dH_2_O and stained with 600 µL 0.1% crystal violet at ambient temperature, shielded from light for 10 min. Wells were then rinsed again 3 times with sterile dH_2_O and air-dried for at least 30 min before images were taken against a white backdrop. Images of stained biofilms from each treatment were processed in ImageJ, by conversion to 8-bit black and white images, followed by auto-local thresholding, using the Phansalker method [69]. ImageJ particle analysis was run on each image, with a set scale, based on inner well diameter, to obtain particle count greater than 0.05 mm^2^ per well, whereby 0.05 mm^2^ represents the cut-off threshold value that is recommended by the software.

### 4.6. Statistical Analysis

AFM experiments were performed twice, with two replicates each. AFM raw height data were collected to determine surface roughness. Biofilm quantification experiments were performed two times, with five replicates each per treatment. Biofilm staining experiments for imaging were performed three times, with two replicates each, per liposome-associated treatments, as well as solvent controls and four replicates for HBD2 only associated treatment and controls. Differences in the number of particles above 0.05 mm^2^ were determined for each sample for statistical significance. One-way ANOVA with Tukey’s post hoc analysis was used to determine statistical significance of observed surface roughness differences and biofilm particle analysis. Welch’s ANOVA with Dunnet’s T3 post hoc analysis was used to determine statistical significance of biofilm quantification, due to high unequal variance of the data. Data were graphed GraphPad Prism version 9.2.0 and R Studio 1.4.1106 and statistically analyzed with GraphPad Prism 9.2.0.

## 5. Conclusions

In summary, we found that the treatment of PA with CL-PL alone and in combination with HBD2 resulted in significantly increased bacterial surface roughness and a loss of extracellular structures, emanating from the bacterial cell, as determined by AFM studies. Light microscopy imaging of crystal violet-stained biofilms revealed that HBD2 combined with CL-PL effected a significantly increased number of biofilm particles greater than 0.05 mm^2^, consistent with a break-up of the microbial mat and increased dispersion. These data suggest that the antimicrobial lipid cholesteryl linoleate effect structural changes on the bacterial surface that interfere with biofilm formation, and these effects are further enhanced by HBD2. This may lay the groundwork for the generation of novel approaches in combatting biofilm-related infections in cystic fibrosis and other conditions.

## Data Availability

The data presented in this study are available in the supplementary material for this article.

## Supplementary Materials

The following are available online at https://www.mdpi.com/article/10.3390/antibiotics10111279/s1, Table S1: data file for Figure 2 (AFM Rq values); Table S2: data file for Figure 4 (biofilm quantification) and Figure 5 (biofilm particle measurement).
